# Supplementary material for: Design of synthetic human gut microbiome assembly and butyrate production
Source: Nat Commun. 2021 May 31;12:3254. doi: 10.1038/s41467-021-22938-y (PMC8166853; doi:10.1038/s41467-021-22938-y)
Supplement: Supplementary file 1 — Supplementary Information [file 41467_2021_22938_MOESM1_ESM.pdf]

Supplementary Figures for

Design of synthetic human gut microbiome assembly and butyrate production

Ryan L. Clark[1], Bryce M. Connors[1,2], David M. Stevenson[3], Susan E. Hromada[1,3], Joshua J. Hamilton[1], Daniel Amador-Noguez[3] & Ophelia S. Venturelli[1,2,3]*

[1]Department of Biochemistry, University of Wisconsin-Madison, Madison, WI 53706
[2]Department of Chemical & Biological Engineering, University of Wisconsin-Madison, Madison, WI 53706
[3]Department of Bacteriology, University of Wisconsin-Madison, Madison, WI 53706

*To whom correspondence should be addressed: venturelli@wisc.edu

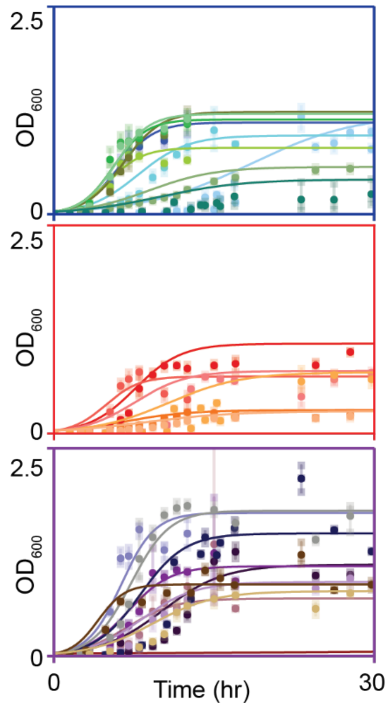

Supplementary Figure 1. Monospecies growth dynamics. Time-series measurements of OD_{600} for single species. Transparent symbols indicate biological replicates (n=2-3) connected to the corresponding mean (solid symbols) by transparent lines. Solid lines represent the generalized Lotka-Volterra (gLV) model fit to the data. Each plot shows the growth curves for species within the Bacteroidetes (top), Actinobacteria/Proteobacteria (middle) or Firmicutes (bottom) phylum. Data point colors correspond to the species names in **Figure 1c**.

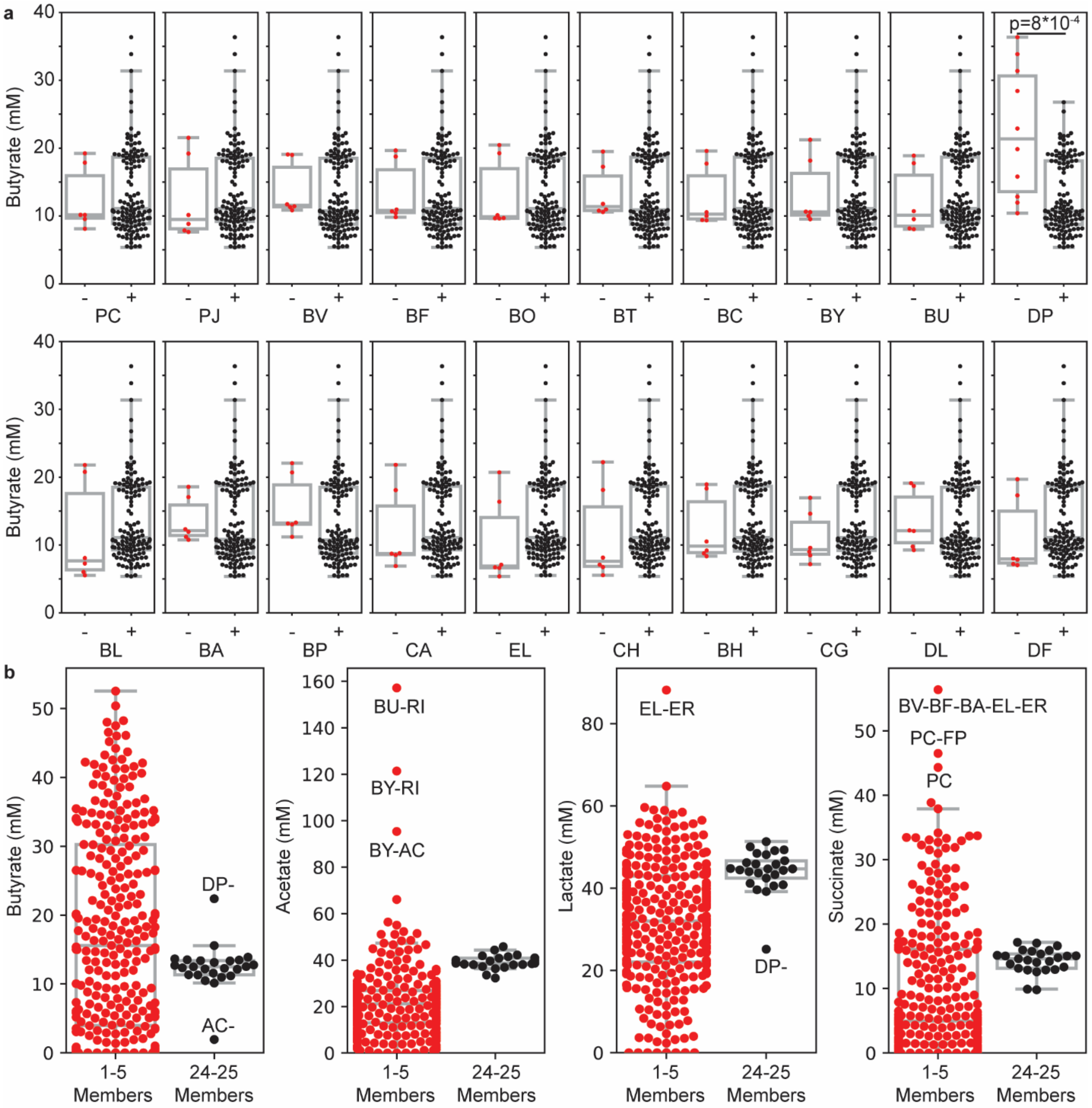

Supplementary Figure 2. Metabolite concentrations at different levels of species richness. **(a)** Categorial scatter plots of butyrate concentrations in single-species deletion communities (24-25 members). Each subplot shows a comparison of all 24-25 member communities that include all 5 butyrate producers with (+) or without (-) the indicated species. Each data point indicates a biological replicate of a community (n=6 for each community). *Desulfovibrio piger* (DP) is the only species with a statistically significant difference using a Kruskal-Wallis test, $p<0.05$ considered significant (p-value indicated in figure for DP). **(b)** Categorial scatter plots of butyrate, acetate, lactate and succinate concentrations in low species richness communities (1-5 members) versus single-species deletion communities (24-25 members). Each datapoint indicates the mean organic acid concentration for each community (n=2-7 biological replicates for each community, exact values in source data). For all boxplots, center line represents the median, box represents the quartiles, and whiskers extend to the entire range of the distribution excluding outliers (those points outside 1.5x the interquartile range).

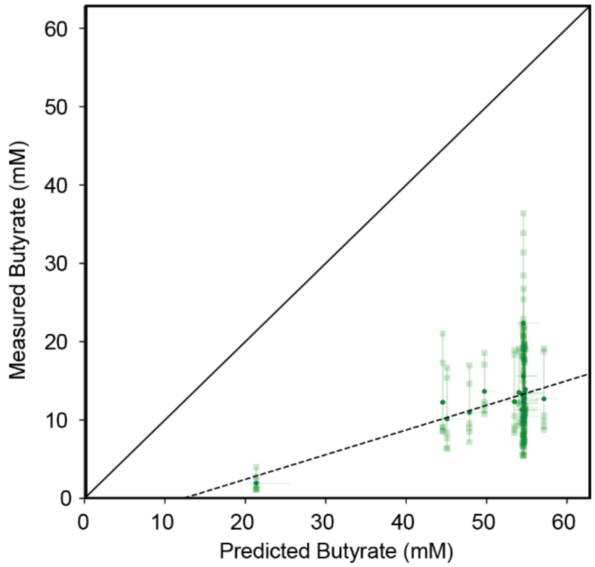

Supplementary Figure 3. Butyrate predictions of 24-25 species communities for model M1. Scatter plot of predicted butyrate versus measured butyrate using model M1 for 24-25 species communities. Biological replicates (n=2-7 biological replicates for each community, exact values in source data) are indicated by transparent squares connected to the corresponding mean (y-axis center), represented as the large data point. Prediction error bars (x-axis) indicate the 60% confidence interval of the predicted butyrate distribution with the median as the center.

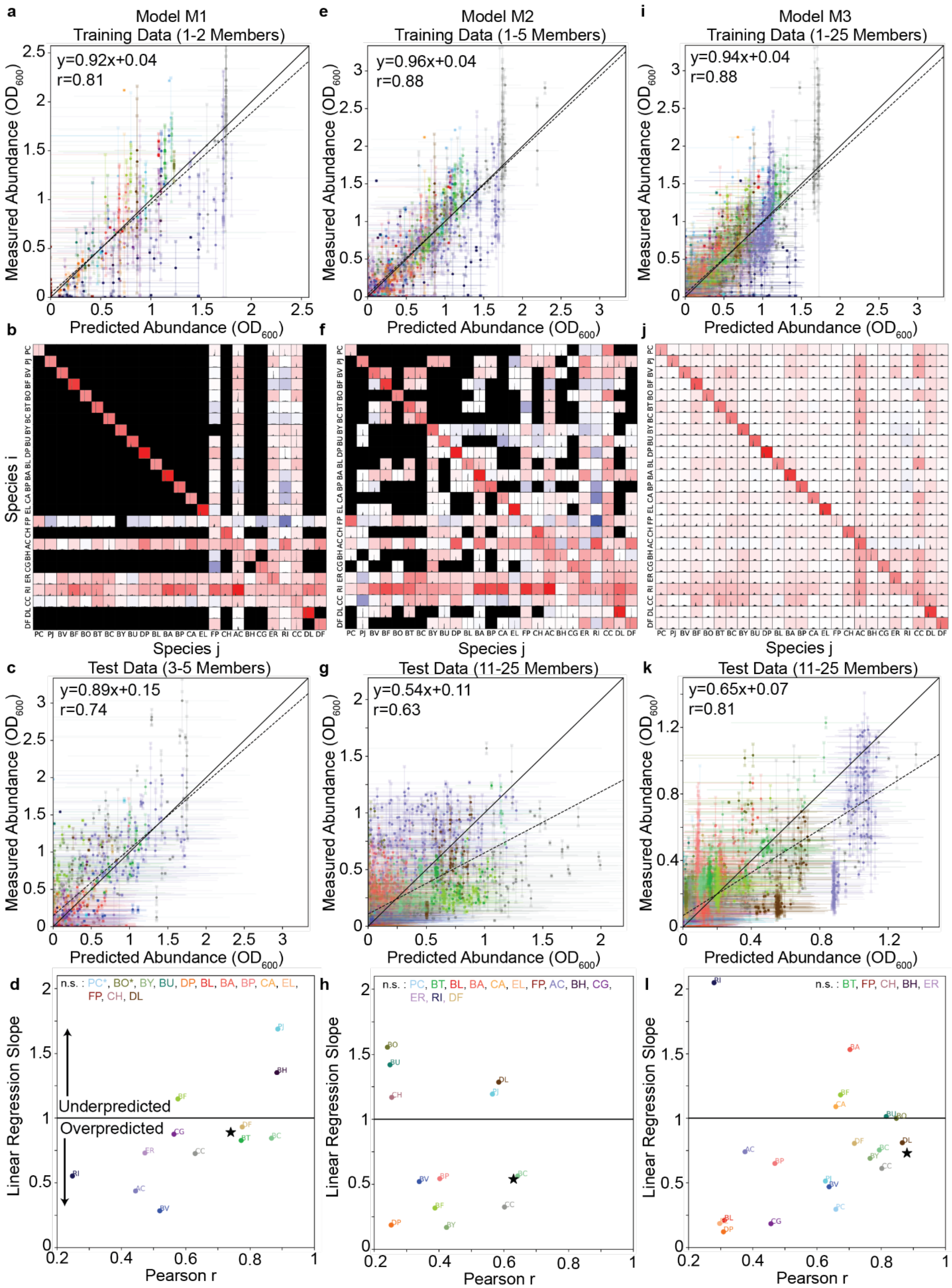

a
Model M1
Training Data (1-2 Members)
y=0.92x+0.04
r=0.81
e
Model M2
Training Data (1-5 Members)
y=0.96x+0.04
r=0.88
i
Model M3
Training Data (1-25 Members)
y=0.94x+0.04
r=0.88
Measured Abundance (OD600)
Predicted Abundance (OD600)
b
f
j
Species i
Species j
PC PJ BV BF BO BT BC BY BU DP BL BA BP CA EL FP CH AC BH CG ER RI CC DL DF
c
Test Data (3-5 Members)
y=0.89x+0.15
r=0.74
g
Test Data (11-25 Members)
y=0.54x+0.11
r=0.63
k
Test Data (11-25 Members)
y=0.65x+0.07
r=0.81
d
n.s. : PC*, BO*, BY, BU, DP, BL, BA, BP, CA, EL, FP, CH, DL
Underpredicted
Overpredicted
h
n.s. : PC, BT, BL, BA, CA, EL, FP, AC, BH, CG, ER, RI, DF
l
n.s. : BT, FP, CH, BH, ER
Linear Regression Slope
Pearson r

Supplementary Figure 4. Prediction of community dynamics using the generalized Lotka-Volterra (gLV) model. (a,e,i) Scatter plots of predicted versus measured species abundance for the training data for models M1-M3. Solid data points at the center indicate the mean measured abundance (y-axis) and median predicted abundance (x-axis) for each species in each community. Transparent data points indicate biological replicate measurements (n=2-7 depending on the community, exact values in source data) and are connected to the corresponding mean values with lines. Horizontal error bars indicate the 60% confidence interval of the model prediction distribution. Colors correspond to legend in **Figure 1c.** Dashed line indicates the linear regression between the mean measured abundances and the median predicted abundances and r represents the Pearson correlation coefficient (two-sided). **(b,f,j)** Heatmaps of model parameters a_{ij}, which quantifies the impact of species *j* on the growth rate of species *i*. Color of the square indicates the median parameter value (red is negative, blue is positive). Histograms within each subplot indicate the distribution of parameter values from the inference analysis. Solid black subplots indicate pairs that were not present in the corresponding training data. **(c,g,k)** Scatter plots of predicted versus measured species abundance for the test data (i.e. communities not included in the training set). Same descriptions as **a,e,i**. **(d,h,l)** Pearson correlation (two-sided) versus linear regression slope for predicted versus measured abundances of each species for the corresponding test data. Black star indicates the statistics for all species together as in **c,g,k**. Species listed after n.s. had Pearson correlations (two-sided) with p>0.05, negative Pearson correlation coefficients, or slope>10. Species indicated with * in **d** had less than three observations in the test data.

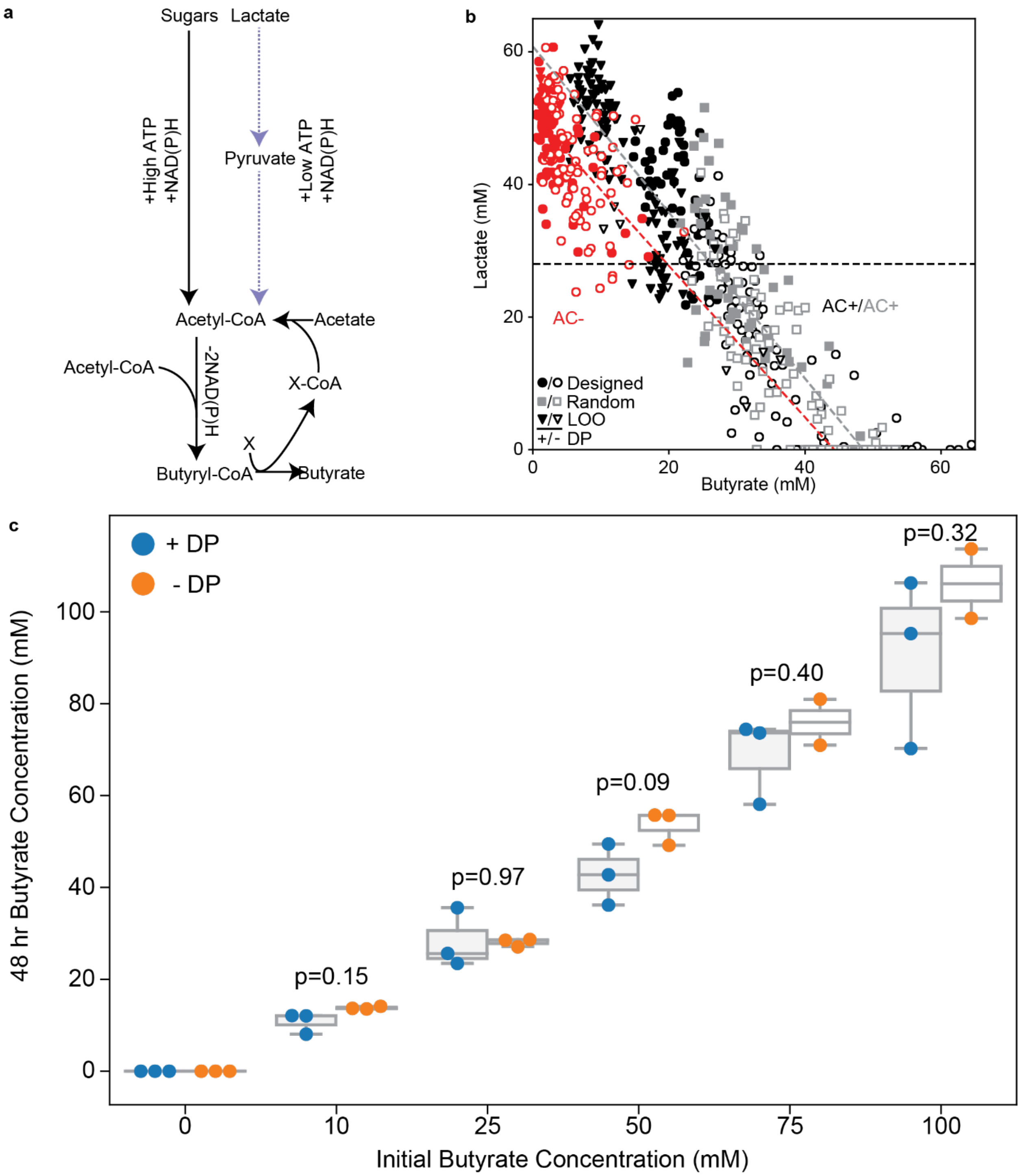

Supplementary Figure 5. Mechanistic investigations of production interactions involving conversion of lactate to butyrate and lack of butyrate consumption by *D. piger*. (a) Metabolic pathways for the transformation of sugars, acetate, and lactate into butyrate. Conversion of sugars or lactate to acetyl-CoA generates ATP and NAD(P)H, with higher ATP production per NAD(P)H from sugars. NAD(P)H is oxidized through conversion of acetyl-CoA to butyryl-CoA. Many substrates (X) can be used to exchange CoA between acetate and/or butyrate. In our system, AC has the unique capability to utilize the lactate conversion pathway (purple dashed arrows). **(b)** Scatter plot of butyrate concentration versus lactate concentration for complex

communities (>10 species). Each data point indicates a biological replicate of a community. Grey dashed line indicates the linear regression for communities containing AC (y=-1.3x+61, Pearson correlation two-sided r=-0.91, $p=5*10^{-182}$), red dashed line indicates the linear regression for communities lacking AC (y=-1.1x+51, Pearson correlation two-sided r=-0.56, $p=8*10^{-16}$) and black horizontal dashed line indicates initial concentration of lactate in the media (28 mM). **(c)** Categorial scatter plot of butyrate concentrations in the presence of DP cultured for 48 hours in media supplemented with the indicated concentration of sodium butyrate (blue) or in a media control incubated for the same period (orange). The p-values were computed using a student's t-test (two-sided, unequal variance). For boxplots, center line represents the median, box represents the quartiles, and whiskers extend to the entire range of the distribution.

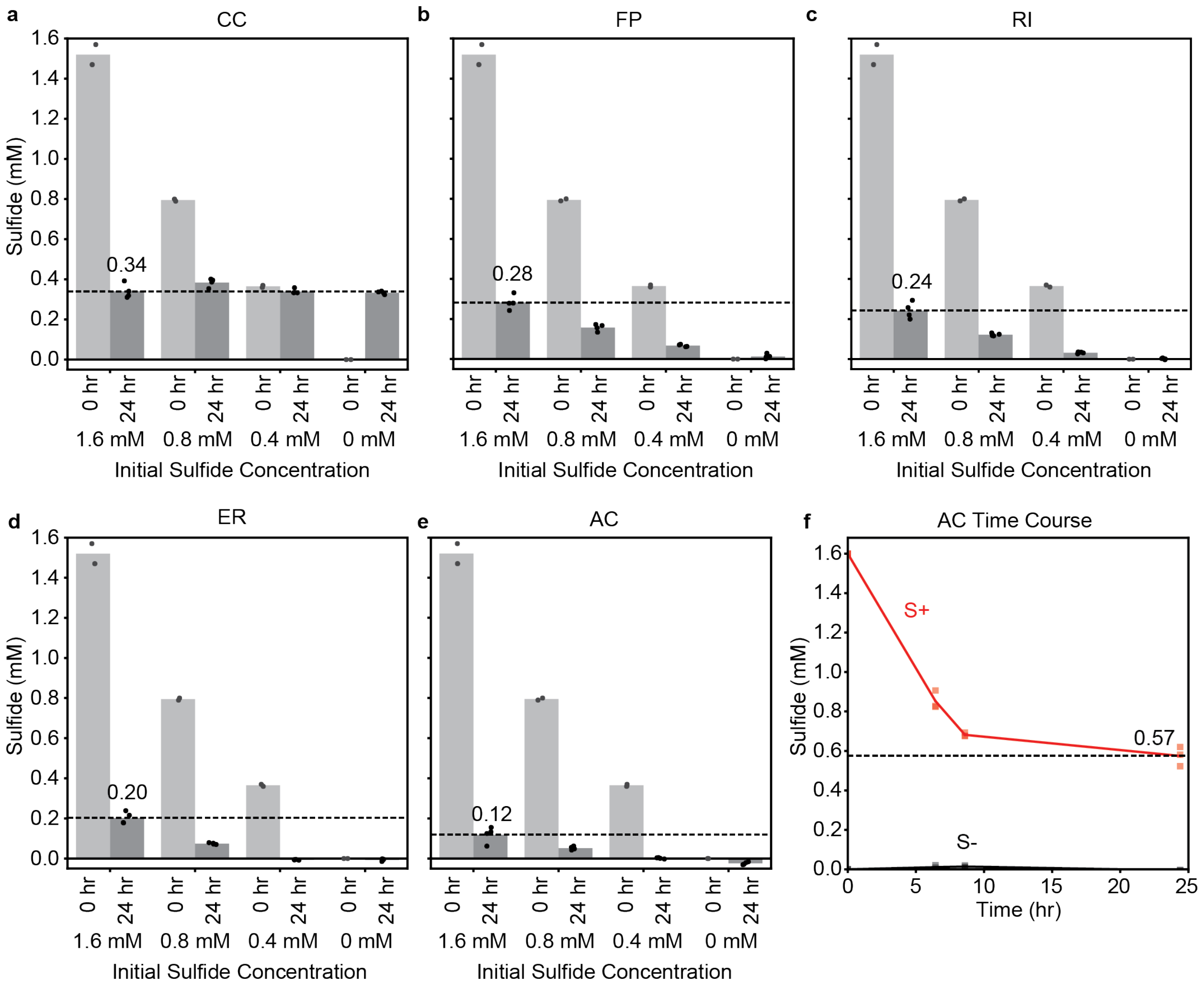

Supplementary Figure 6. Sulfide concentrations at different time points. (a-e) Sulfide concentrations measured at 0 hr and 24 hr for the indicated species for the experiment in **Figure 3f**. Bars indicate the mean of replicates and data points show individual measurements (0 hr, n=2 technical replicates; 24 hr, n=4 biological replicates). Dashed line and numerical value indicate the mean concentration at 24 hr for the 1.6 mM condition for comparison across species. **(f)** Time course of sulfide concentrations for the experiment in **Figure 3f, inset**. Data points indicate individual measurements (n=3 biological replicates) and the line follows the mean. Dashed line and numerical value indicate the mean concentration at 24 hr for the 1.6 mM sulfide condition for comparison to **(a-e)**.

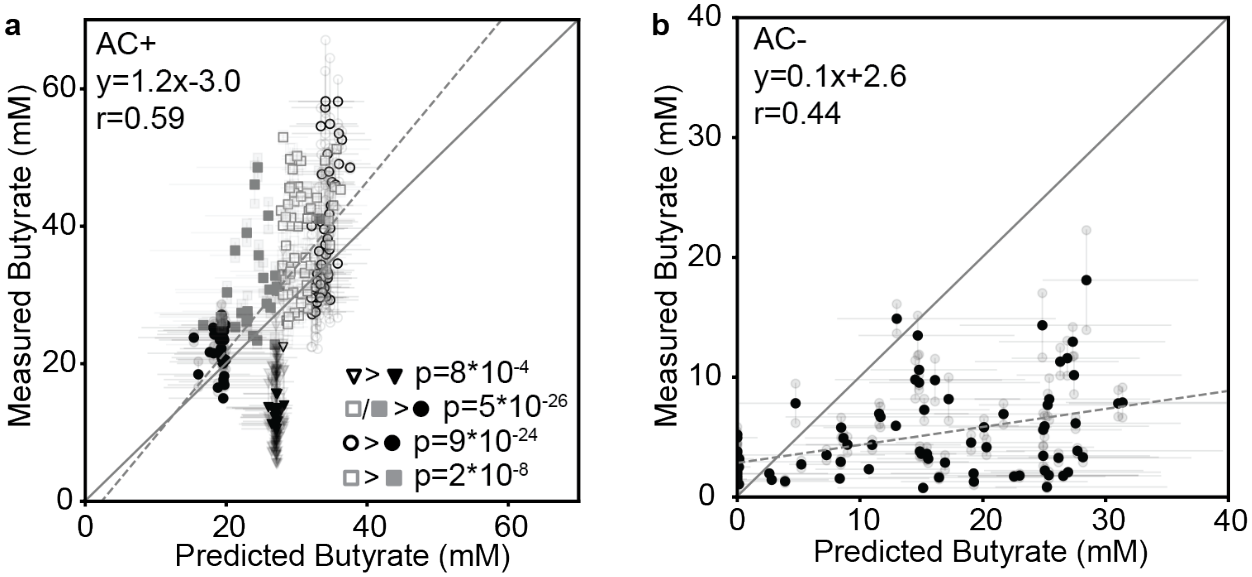

Supplementary Figure 7. Butyrate predictions for model M2. (a) Scatter plot of predicted versus measured butyrate concentration for communities in **Figure 3a**. Transparent squares indicate biological replicates (n=1-2 for circles and squares, 3-7 for triangles, depending on community, exact values in source data) and are connected to the corresponding mean values by transparent lines. Data points denote the median with error bars spanning the 60% confidence interval. Solid line indicates x=y. Dashed line indicates linear regression of median prediction versus mean measurement, with slope and intercept indicated in top left, with Pearson correlation r ($p=1.3*10^{-18}$, two-sided). Legend indicates statistically significant differences in measured butyrate between populations of communities (Kruskal-Wallis test). **(b)** Scatter plot of predicted versus measured butyrate for communities in **Figure 3b**. Transparent data points indicate biological replicates (n=1-2 depending on the community, exact values in source data) and are connected to the corresponding mean by transparent lines. Data points represent the median with error bars spanning the 60% confidence interval. Solid grey line indicates x=y. Dashed line indicates linear regression of median prediction versus mean measurement, with slope and intercept indicated in top left, with Pearson correlation r ($p=5.3*10^{-5}$, two-sided).

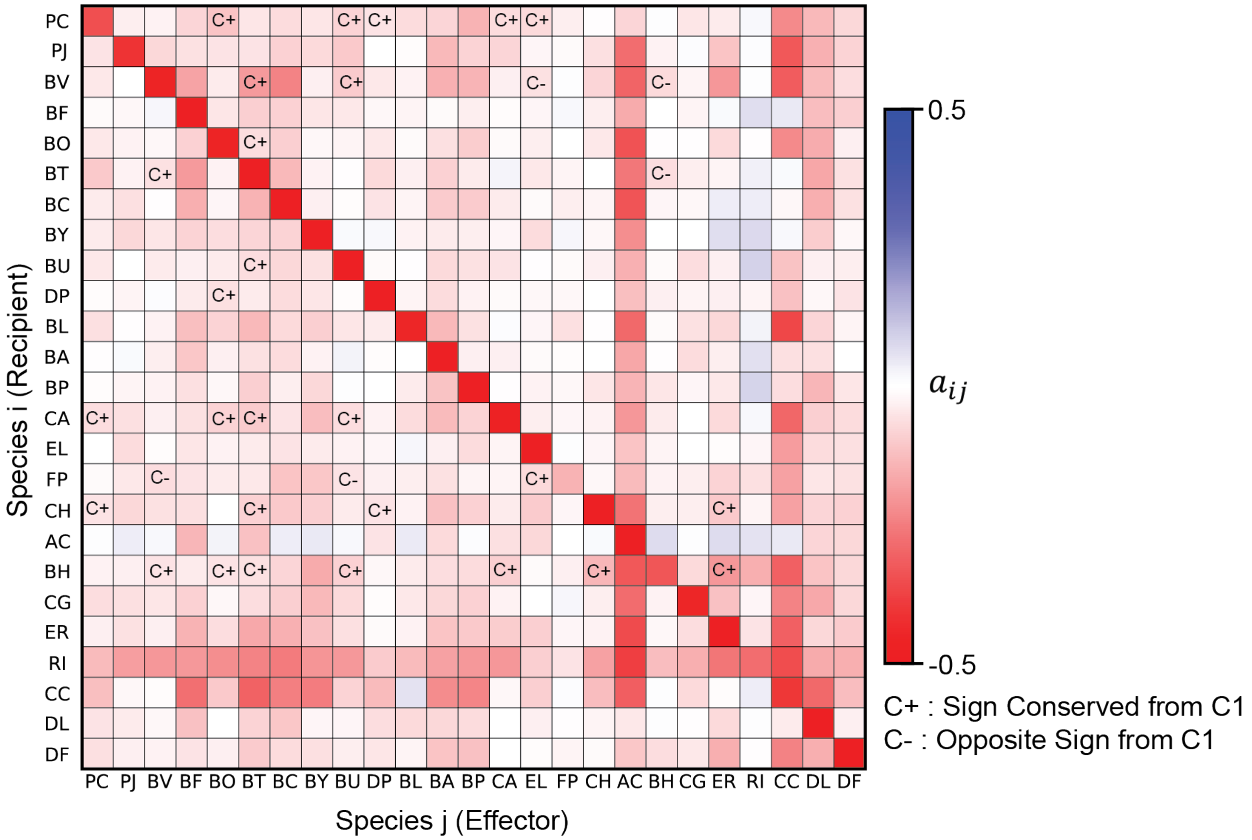

Supplementary Figure 8. Heatmap of inferred gLV parameters for the M3 model. Heatmap of the median value of the inter-species interaction coefficients (a_{ij}) for the M3 gLV model. Inter-species interactions included in the model community C1 from (Venturelli et al., Mol. Sys. Bio., 2018)[6] are annotated with C+ or C- if interactions from both models had magnitudes greater than 0.05 hr^{-1} and had the same or opposite sign, respectively.

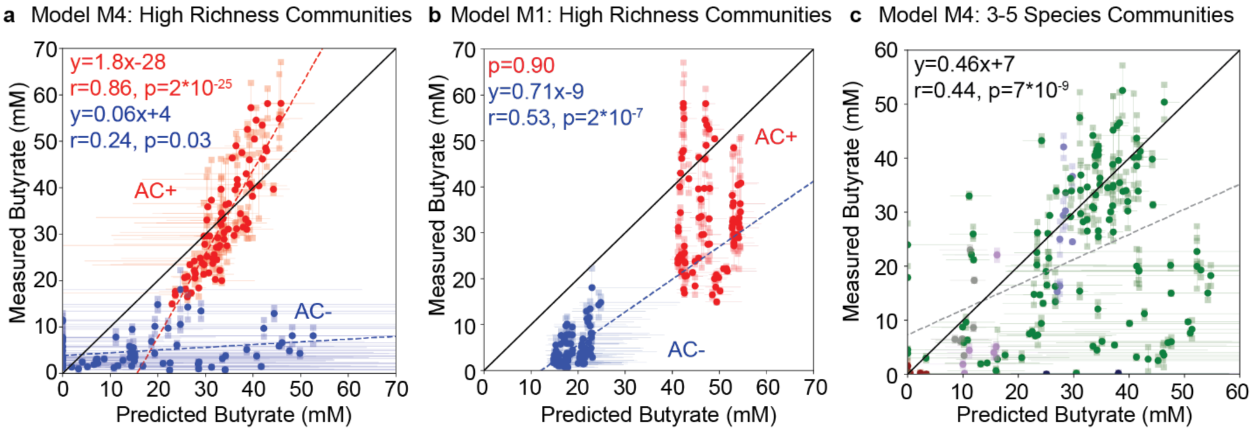

Supplementary Figure 9. Comparison of the predictive capability of models trained on pairwise communities (M1) versus high richness communities (M4). Scatter plot of predicted versus measured butyrate concentration for **(a)** Model M4 predicting the designed communities in **Figure 3a-b** (i.e. excluding random and 24-25 member communities); **(b)** Model M1 predicting the same communities as **(a)** (note that p-value was larger than 0.05 for AC+ in this case, thus we did not include linear regression statistics); and **(c)** Model M4 predicting the 3-5 species communities from **Figure 2c**. Transparent squares indicate biological replicates (n=1-2 for **(a)** and **(b)**, n=1-5 for **c**, depending on community, exact values in source data) and are connected to the corresponding mean values by transparent lines. Data points denote the median with error bars spanning the 60% confidence interval. Solid line indicates x=y. Dashed line indicates linear regression of median prediction versus mean measurement. Colors in **(c)** indicate which butyrate-producer(s) were present in the community as in **Figure 2a**. All indicated statistics are for Pearson correlation (two-sided).

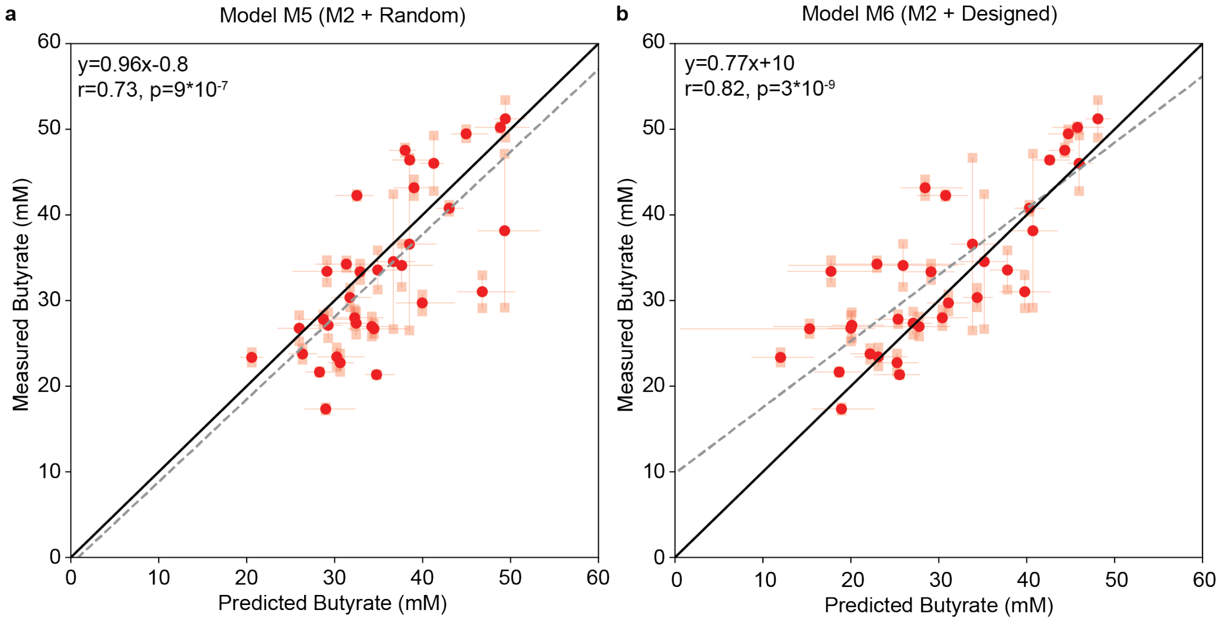

Supplementary Figure 10. Comparing models trained on random versus designed community data. Scatter plot of predicted versus measured butyrate concentration for **(a)** Model M5 trained on data from M2 plus 80% of the random 5BP communities, and **(b)** Model M6 trained on data from M2 plus 80% of the designed 5BP communities. The validation set consisted of 20% of communities from the designed and random 5BP communities excluded from training (same validation set in both plots). Transparent square data points indicate biological replicates (n=1-2) and are connected to the corresponding mean values by transparent lines. Data points denote the median with error bars spanning the 60% confidence interval. Solid line indicates x=y. Dashed line indicates linear regression of median prediction versus mean measurement. All indicated statistics are for Pearson correlation (two-sided).

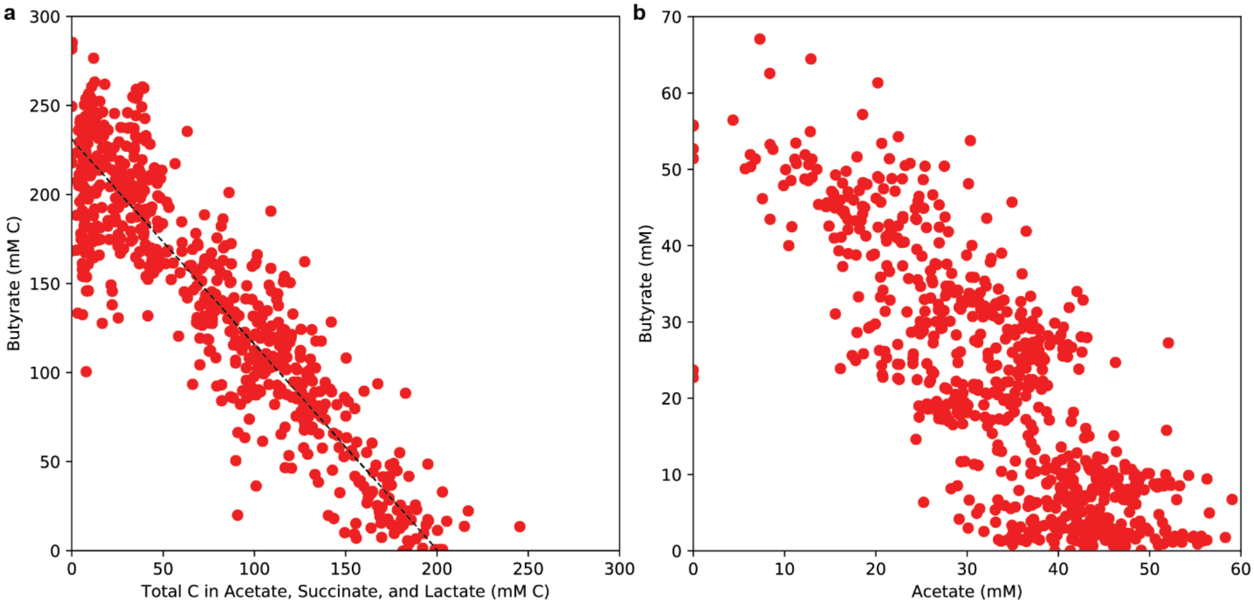

Supplementary Figure 11. Trade-offs in organic acid production. (a) Scatter plot of total carbon in acetate, succinate, and lactate versus carbon in butyrate for communities with greater than 10 species. Each point indicates a biological replicate of a community. Dashed line indicates the linear regression (y=-1.15x+231, Pearson correlation two-sided r=-0.93, $p=7*10^{-293}$). **(b)** Scatter plot of butyrate concentration versus acetate concentration for communities with greater than 10 species. Each point indicates a biological replicate of a community.

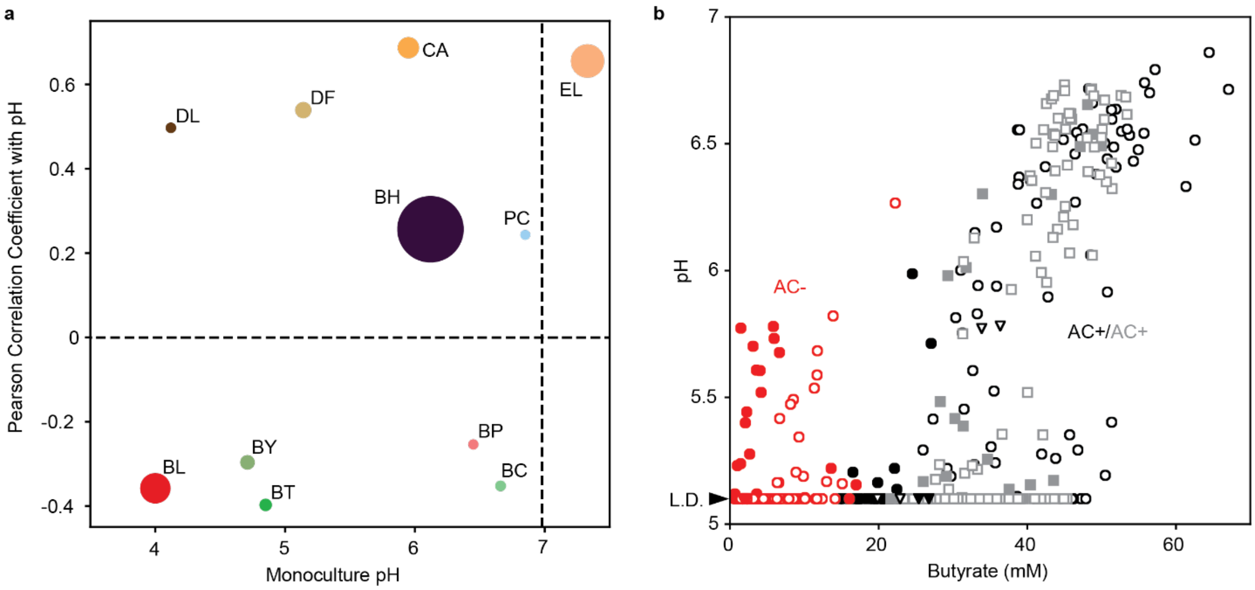

Supplementary Figure 12. Relationship between environmental pH of monocultures and correlation between species abundance and environmental pH. (a) Scatter plot of environmental pH of monospecies cultures at 48 hours versus the Pearson correlation coefficient (two-sided) of absolute species abundance with environmental pH at 48 hr in communities containing greater than 10 species. The size of the data points corresponds to the magnitude of the slope of the linear regression. Only those species with a statistically significant Pearson correlation ($p<0.05$, two-sided) are shown. Vertical dashed line indicates the initial environmental pH. **(b)** Scatter plot of butyrate concentration versus environmental pH for high richness communities. Each data point indicates a biological replicate of a community.

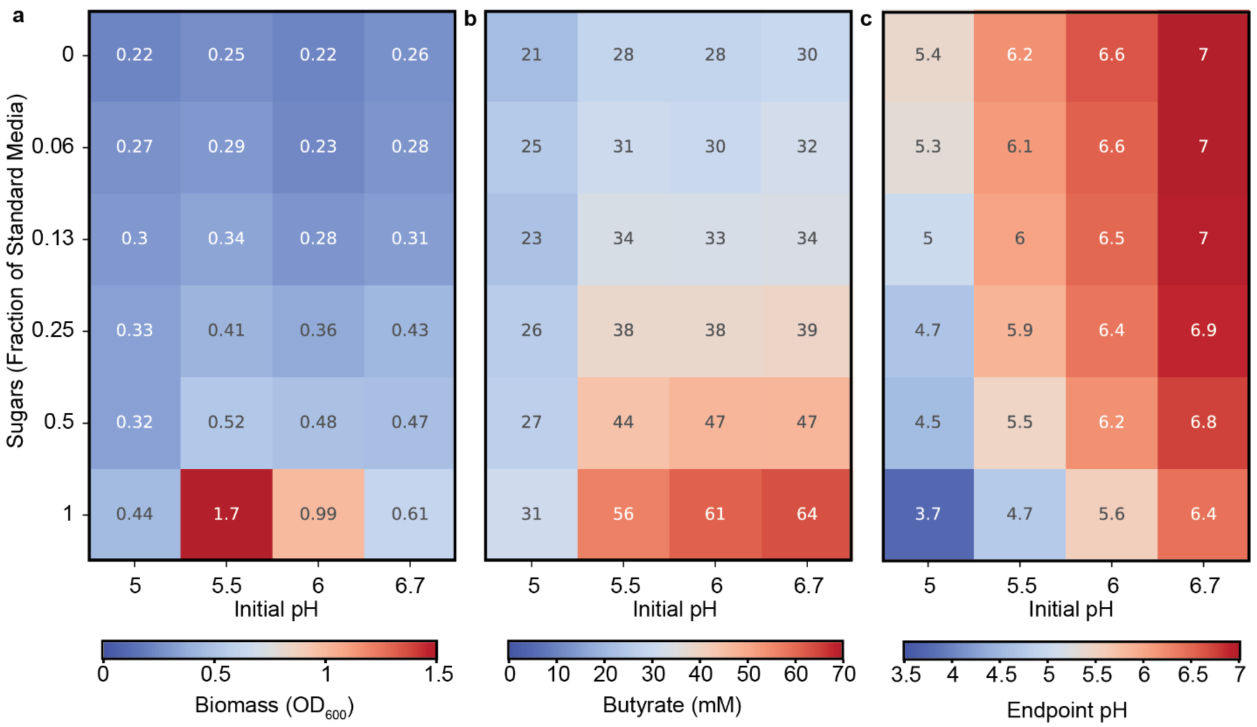

Supplementary Figure 13. Growth of *A. caccae*, butyrate production, and environmental pH modification across a range of sugar concentrations and initial environmental pH values. Heatmaps indicate average of three biological replicates for measurements of **(a)** AC biomass, **(b)** butyrate concentration, and **(c)** endpoint environmental pH.
