# Supplementary material for: Design of synthetic human gut microbiome assembly and butyrate production
Source: Nat Commun. 2021 May 31;12:3254. doi: 10.1038/s41467-021-22938-y (PMC8166853; doi:10.1038/s41467-021-22938-y)
Supplement: Supplementary file 8 — Reporting Summary [file 41467_2021_22938_MOESM8_ESM.pdf]

nature research

Corresponding author(s): Ophelia S. Venturelli

Last updated by author(s): Mar 28, 2021

Reporting Summary

Nature Research wishes to improve the reproducibility of the work that we publish. This form provides structure for consistency and transparency in reporting. For further information on Nature Research policies, see our Editorial Policies and the Editorial Policy Checklist.

Statistics

For all statistical analyses, confirm that the following items are present in the figure legend, table legend, main text, or Methods section.

n/a	Confirmed	
☐	☒	The exact sample size (*n*) for each experimental group/condition, given as a discrete number and unit of measurement
☐	☒	A statement on whether measurements were taken from distinct samples or whether the same sample was measured repeatedly
☐	☒	The statistical test(s) used AND whether they are one- or two-sided<br>*Only common tests should be described solely by name; describe more complex techniques in the Methods section.*
☒	☐	A description of all covariates tested
☒	☐	A description of any assumptions or corrections, such as tests of normality and adjustment for multiple comparisons
☐	☒	A full description of the statistical parameters including central tendency (e.g. means) or other basic estimates (e.g. regression coefficient) AND variation (e.g. standard deviation) or associated estimates of uncertainty (e.g. confidence intervals)
☐	☒	For null hypothesis testing, the test statistic (e.g. *F*, *t*, *r*) with confidence intervals, effect sizes, degrees of freedom and *P* value noted<br>*Give P values as exact values whenever suitable.*
☐	☒	For Bayesian analysis, information on the choice of priors and Markov chain Monte Carlo settings
☒	☐	For hierarchical and complex designs, identification of the appropriate level for tests and full reporting of outcomes
☐	☒	Estimates of effect sizes (e.g. Cohen's *d*, Pearson's *r*), indicating how they were calculated

Our web collection on statistics for biologists contains articles on many of the points above.

Software and code

Policy information about availability of computer code

Data collection	Sequencing data were collected on an Illumina MiSeq instrument (MiSeq Control Software 3.1.0.13) and the BaseSpace Sequencing Hub's FASTQ Generation Program was used to generate FASTQ files for further analysis with custom Bioinformatics workflows (using PEAR v0.9.10 and tools in mothur v1.40.5, more details in the Methods). Robotic automation methods using Tecan Freedom EVO for generating microbial community inocula and normalizing genomic DNA for the NGS workflow were programmed using the worklist function within the Tecan Freedom EVOWare software version 2.7. Optical density measurements with the Tecan F200 plate reader were carried out with the standard Tecan i-Control software version 3.8.2.0. Absorbance measurements with the Tecan Spark plate reader were carried out with the standard SparkControl software version 2.3. HPLC Peak integration was performed using either the ThermoFisher Chromeleon 7 or Shimadzu LabSolutions version 5.89 software packages.
Data analysis	Please see the Software README in our Github Repository (https://github.com/RyanLincolnClark/DesignSyntheticGutMicrobiomeAssemblyFunction) for a comprehensive description of the custom scripts used for analysis in this work. These scripts utilize MATLAB R2016a or 2018a, Python 3, and Julia v0.6.4 (specification files containing the versions of all packages used in Python 3 and Julia v0.6.4 are available in the Git Repository).

For manuscripts utilizing custom algorithms or software that are central to the research but not yet described in published literature, software must be made available to editors and reviewers. We strongly encourage code deposition in a community repository (e.g. GitHub). See the Nature Research guidelines for submitting code & software for further information.

Data

Policy information about availability of data

All manuscripts must include a data availability statement. This statement should provide the following information, where applicable:

- Accession codes, unique identifiers, or web links for publicly available datasets
- A list of figures that have associated raw data
- A description of any restrictions on data availability

Data supporting the findings described in this work are available from the corresponding author upon request. The processed data for all community experiments and simulation results are available in the Github Repository described in our Code Availability statement (https://github.com/RyanLincolnClark/DesignSyntheticGutMicrobiomeAssemblyFunction). The Illumina Sequencing data are available via Zenodo at https://doi.org/10.5281/zenodo.4642238.

Field-specific reporting

Please select the one below that is the best fit for your research. If you are not sure, read the appropriate sections before making your selection.

☒ Life sciences ☐ Behavioural & social sciences ☐ Ecological, evolutionary & environmental sciences

For a reference copy of the document with all sections, see nature.com/documents/nr-reporting-summary-flat.pdf

Life sciences study design

All studies must disclose on these points even when the disclosure is negative.

Sample size	Sample sizes were chosen based on limitations of experimental throughput as increased number of biological replicates would have reduced the number of possible different communities that could be observed. We chose a minimum of 2 biological replicates (for complex communities in our validation set) and some sample types have up to 7 biological replicates (such as the full community, which was repeated in most experiments as a control for consistency between experimental days). These number of replicates were sufficient for us to quantify biological variability across the large number of communities we measured. All statistical tests on trends across communities were performed on groups of communities, rather than comparing 2 specific communities for instance, so the number of different communities determined our statistical power, and was sufficient to observe the indicated trends.
Data exclusions	Individual samples were excluded from the analysis if they were determined to be contaminated by species not intended to be in the sample. Contamination was determined to be any species >1% of the sample. This cutoff was chosen based on the ratio of typical background to typical read depth for our samples.
Replication	The experiments of various communities were performed in 9 different batches and the full community was included in each of these batches to quantify variation across the different experimental batches. The 24-species "leave-one-out" communities were also repeated in three of the batches. The variance of these replicated communities can be seen in Figures 2a, 3c,d, and 4a. The sulfide titration experiment was replicated on 3 separate occasions with slight differences in experimental approach while optimizing the procedure to limit loss of H_2S to the gas phase and agreed at least qualitatively with the data shown on each occasion.
Randomization	This study was aimed at designing communities, so we did not randomize our experimental design, with the exception of the Random Community set described in Figure 3. We did not randomize, rather all biological replicates were processed in separate batches, so any variation due to external factors would be captured in the variance between biological replicates of each treatment.
Blinding	For the community design experiments, the HPLC analysis was carried out in a blinded manner. The samples were labeled only with a numerical identifier and given to a separate researcher for measurement. Otherwise, while the experimental allocations were known to the experimental investigator because the same individual designed the experiments, the large number of samples were labeled using a code and thus were practically unknown prior to analysis of the final aggregated data.

Reporting for specific materials, systems and methods

We require information from authors about some types of materials, experimental systems and methods used in many studies. Here, indicate whether each material, system or method listed is relevant to your study. If you are not sure if a list item applies to your research, read the appropriate section before selecting a response.

Materials & experimental systems

n/a	Involved in the study
[x]	[ ] Antibodies
[x]	[ ] Eukaryotic cell lines
[x]	[ ] Palaeontology and archaeology
[x]	[ ] Animals and other organisms
[x]	[ ] Human research participants
[x]	[ ] Clinical data
[x]	[ ] Dual use research of concern

Methods

n/a	Involved in the study
[x]	[ ] ChIP-seq
[x]	[ ] Flow cytometry
[x]	[ ] MRI-based neuroimaging
