# Supplementary material for: Design of synthetic human gut microbiome assembly and butyrate production
Source: Nat Commun. 2021 May 31;12:3254. doi: 10.1038/s41467-021-22938-y (PMC8166853; doi:10.1038/s41467-021-22938-y)
Supplement: Supplementary file 10 — Description of Additional Supplementary Files [file 41467_2021_22938_MOESM10_ESM.pdf]

Title: Supplementary Data 1.
Description: Strain information. Table of strain information for synthetic human gut microbiome community.

Title: Supplementary Data 2.
Description: Descriptions of Model Training Data. Information about datasets used to train models M1-M6.

Title: Supplementary Data 3.
Description: Regression Model Parameters. Inferred regression model parameters for models M1-M3. Only those parameters with magnitude greater than 4 mM are shown.

Title: Supplementary Data 4.
Description: Defined Media Composition. Table of compoonents of the defined media used to culture the synthetic human gut community.

Title: Supplementary Data 5.
Description: Sequencing Library Primers. Table of next-generation sequencing primers used for 16S rRNA gene sequencing.

Title: Supplementary Data 6.
Description: Generalized Lotka-Volterra Model Regularization Parameters. Table of generalized Lotka-Volterra model regularization parameters. The parameter w represents the weighting factor for the time-series monospecies measurements and λ represents the regularization coefficient.
